# A rare case report of epithelioid hemangioendothelioma of the left buttock: Challenges in diagnosis and management

**DOI:** 10.1097/MD.0000000000044137

**Published:** 2025-08-29

**Authors:** Jong Yun Choi, Hee Yang Park, Suk-Ho Moon, Deuk Young Oh, Young-Joon Jun, Jangyoun Choi

**Affiliations:** a Department of Plastic and Reconstructive Surgery, Seoul St. Mary’s Hospital, The Catholic University of Korea College of Medicine, Seoul, South Korea.

**Keywords:** diagnosis, hemangioepithelioma, neoplasm metastasis, recurrence, sarcoma

## Abstract

**Rationale:**

Epithelioid hemangioendothelioma (EHE) is a rare vascular malignancy which poses significant diagnostic and therapeutic challenges due to its variable course and lack of standardized treatment protocols. In this case, the lesion’s benign appearance and ambiguous histology delayed definitive diagnosis.

**Patient concerns:**

A 46-year-old woman presented with a recurrent soft tissue mass in the left buttock. Initial impression with partial biopsy favored a benign neoplasm, complicating the diagnosis.

**Diagnoses:**

Histopathological examination confirmed EHE, characterized by epithelioid endothelial cells and positive immunohistochemical staining for vascular markers.

**Interventions:**

Complete surgical resection was performed, with no adjuvant therapy administered postoperatively.

**Outcomes:**

Surveillance over 24 months revealed no evidence of local recurrence or distant metastasis. Follow-up computed tomography on 18 month revealed no evidence of recurrence.

**Lessons:**

Comprehensive histopathological evaluation are critical for proper diagnosis of EHE. Surgical resection is the first line treatment for localized EHE. Long-term monitoring is essential due to the unpredictable metastatic potential.

## 1. Introduction

Epithelioid hemangioendothelioma (EHE) is an ultrarare vascular sarcoma characterized by epithelioid endothelial cells, whose natural history and treatment are not well defined.^[1,2]^ Histologically, EHE is identified by epithelioid endothelial cells with intracytoplasmic vacuoles and immunoreactivity for vascular markers such as CD31 and FLI-1. First reported in 1982 by Weiss and Enzinger, the term “epithelioid hemangioendothelioma” was coined to describe a vascular tumor originating from vascular endothelial cells and showing features between those of a hemangioma and angiosarcoma.^[3]^

EHE is an extraordinarily rare tumor, with a prevalence of one in 1 million, and the relevant literature is limited to case reports and few retrospective case series. It has an unpredictable clinical behavior ranging from indolent to aggressively malignant.^[4]^ According to reports so far, EHE commonly arises in the lungs, liver, or bone. However, the clinical presentation of EHE is heterogeneous, and any part of the body can be involved.^[1]^ Its diagnosis is confirmed by biopsy and immunostaining for endothelial markers. Unlike most previously reported cases, the present case is notable for a soft tissue recurrence without distant metastasis, which was successfully managed by surgical excision alone. This unique clinical course highlights the importance of individualized management strategies for EHE.

Due to its uncommon presentation and diagnostic ambiguity, we present an extraordinary case of EHE in a 46-year-old female patient who initially presented with a left buttock mass.

## 2. Case report

A 46-year-old female without significant past medical history presented to the dermatology department of our institution, where an excisional biopsy was performed by a dermatologist. Histologically, irregularly shaped spindle cells with fibrotic stroma were observed to proliferate and form nests in small units, and abundant eosinophilic cytoplasm and vacuoles were observed inside the cells, suggestive of a benign spindle cell neoplasm

**Figure 1.**
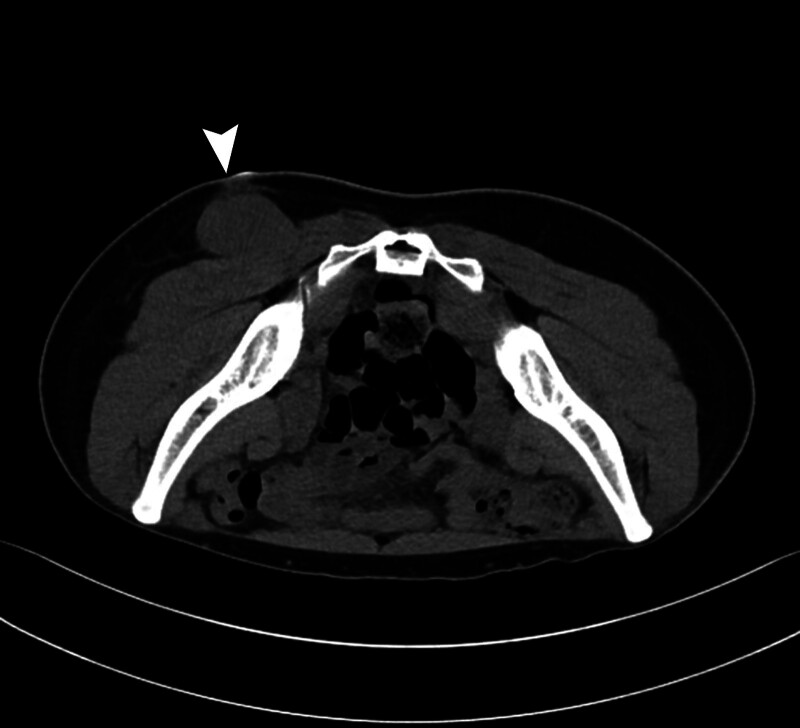
Initial CT imaging showing a 4.5-cm ovoid, dense soft tissue mass at the posterior aspect of the left gluteus maximus muscle, with indistinct borders. CT = computed tomography.

Four months later, the mass was palpated in the same location again, and there was no interval change in size for the following 6 months. The patient visited our department for removal, and the mass was soft, non-tender, and oval-shaped. A preoperative computed tomography scan showed an approximately 4.5-cm-sized dense soft tissue lesion in the subcutaneous layer of the left buttock with diffuse contact with the left gluteal muscle (Fig. 1).

**Figure 2.**
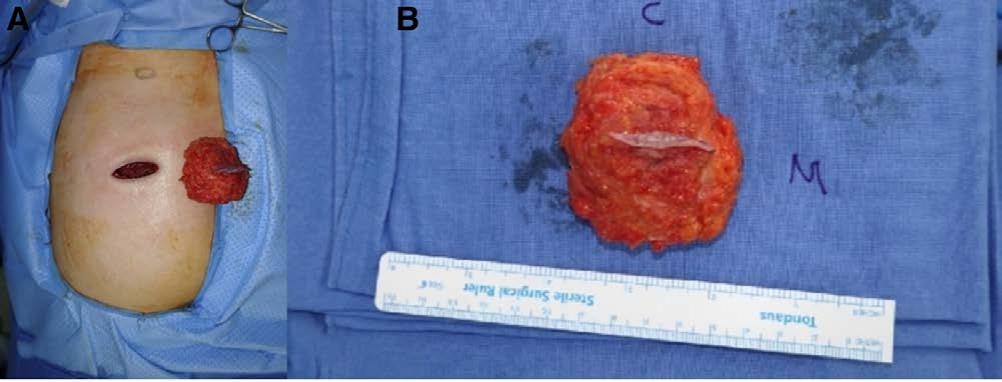
(A) Gross specimen of the tumor after en bloc resection. (B) Close-up photo of the specimen.

**Figure 3.**
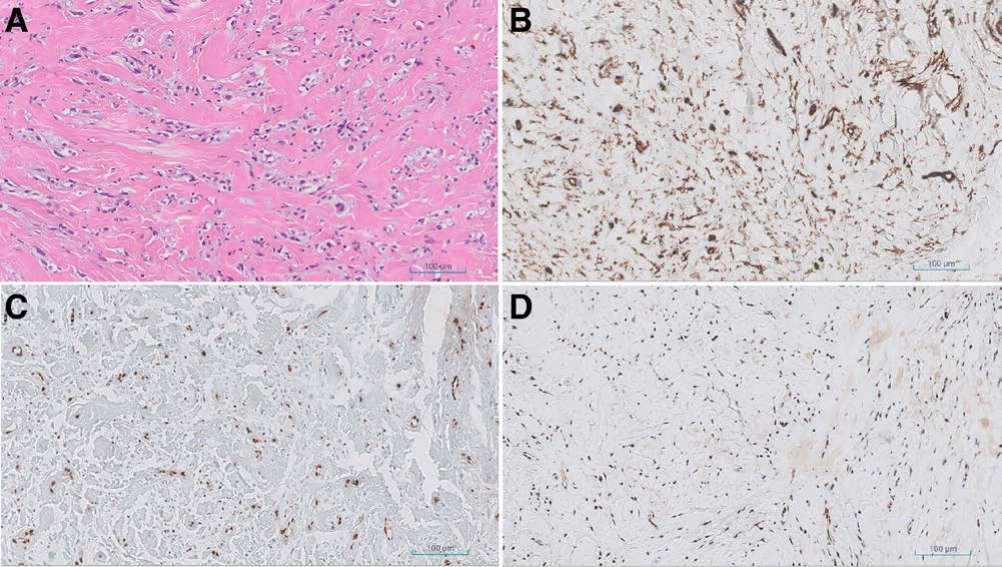
Histologic findings. (A) Hematoxylin and eosin stain showing irregularly shaped spindle cells with fibrotic stroma. (B) Positive staining with CD 31 immunohistochemistry. (C) Positive stain for ERG immunohistochemistry. (D) Positive stain for FLI-1 immunohistochemistry (×100).

En bloc resection of the tumor was performed. A new elliptical excision design incorporating the previous incision was designed. Wide subcutaneous dissection to achieve a safety margin of 1.5 cm was performed. The mass was removed en bloc. On gross histology, the specimen measured 5.5 cm × 5.5 cm × 4.4 cm (Fig. 2). Histologically, irregularly shaped spindle cells with fibrotic stroma on hematoxylin and eosin stain. Immunohistochemistry showed diffuse positivity for CD31, ERG, and FLI-1 (Fig. 3). Based on the gross histological and immunohistochemical stains, the diagnosis of EHE was made. The resection margin was negative in all directions, achieving R0 resection status.

**Figure 4.**
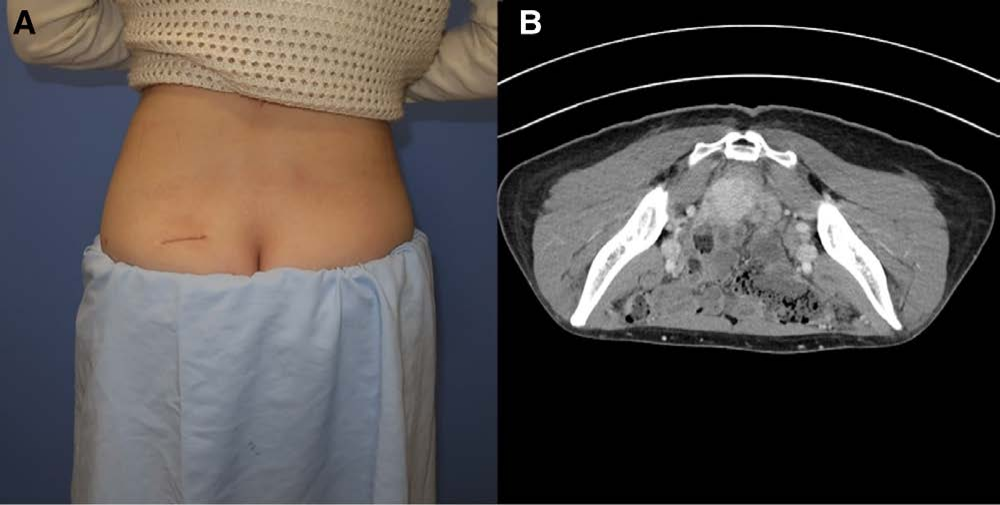
Clinical photograph and CT scan at the 18-month follow-up, demonstrating no evidence of recurrence or complications. CT = computed tomography.

**Figure 5.**
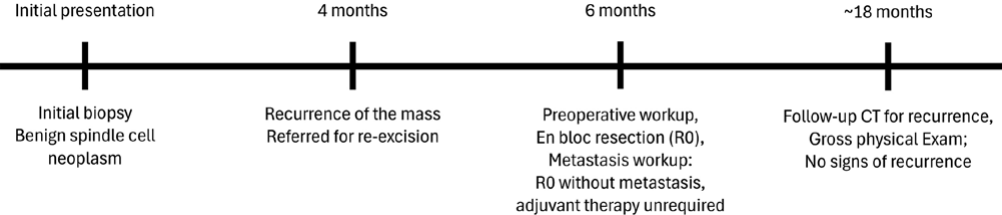
Brief treatment timeline of the case.

For staging purposes, the patient underwent abdominal and chest computed tomography scans in the oncology department, but no metastasis to other organs was observed. Additionally, in consultation with the radiation oncology department, no further radiotherapy was deemed necessary as complete resection was achieved. The patient remained under routine surveillance 18 months postoperatively, with no significant complications. Closed follow-up with 6 months clinical exams, and imaging studies obtained at 18 months follow-up interval demonstrated no evidence of recurrence or notable complications, confirming successful outcome of the treatment (Fig. 4). A timeline of the patient’s clinical course is summarized (Fig. 5). The decision not to administer adjuvant chemotherapy was based on the achievement of complete surgical resection with clear margins (R0 resection), the absence of high-risk histological features, and the lack of evidence supporting the benefit of adjuvant therapy in localized EHE according to current guidelines. The patient expressed relief after receiving an accurate diagnosis following an initial period of uncertainty and recurrence. The absence of recurrence or complications during long-term surveillance allowed her to return to normal life.

All procedures performed in this study were in accordance with the ethical standards of the institutional and/or national research committee and with the Helsinki Declaration (as revised in 2013). Written informed consent was obtained from the patient for publication of this case report and accompanying images. A copy of the written consent is available for review by the editorial office of this journal.

## 3. Discussion

### 3.1. Epidemiology and diagnosis

The present case describes the clinical and histopathological features of an EHE in soft tissue. The incidence of EHE is 0.038/1,00,000 per year, with peak presentation between the fourth and fifth decades of life. Its prevalence is less than 1/10,00,000. Due to this exceedingly rare presentation, there are no solid guidelines for evaluation and treatment.

As mentioned, EHE tends to present in middle age, and it shows a slight preponderance in women. EHE can develop anywhere in the body and manifest in a variety of ways, such as a single lesion, locoregional metastases, or systemic metastases. In the majority of cases, EHE is multifocal or metastatic at diagnosis. When the onset presents as a single lesion, it is typically a single mass in the soft tissues. In the case of metastasis, the lungs, liver, and bone are most highly involved.^[5]^ More than half of patients are asymptomatic, and the initial diagnosis is often incidental. The 3 most frequent symptoms in symptomatic patients are pain (40%), a palpable mass (6–24%), and weight loss (9%).^[6,7]^

For diagnosis, the majority of soft tissue EHEs are solitary, had an uneventful clinical course, and were usually treated with curative surgery. Distant metastases, lymph nodes, pleural involvement, and multifocality all had considerably worse outcomes.^[5]^

### 3.2. Histopathologic features

EHE is histologically recognized by epithelioid endothelial cells exhibiting abundant eosinophilic cytoplasm, intracytoplasmic vacuoles, and a variable fibromyxoid stroma. Immunohistochemical studies consistently show positivity for vascular markers, notably CD31 and CD34, as well as FLI-1.^[4]^ The main differential diagnoses include epithelioid angiosarcoma and epithelioid sarcoma, which displays greater nuclear pleomorphism.^[7]^

In a series of 49 patients with skin or soft tissue EHE who were mainly treated surgically, the disease-specific 5-year survival rate was 81%. Disease-related deaths did not occur in any of the patients with low-risk characteristics (tumor size < 3 cm and < 3 mitotic figures/50 high-powered fields). In patients with a high-risk characteristic (tumor size > 3 cm or > 3 mitotic figures/50 high-powered fields), the 5-year disease-specific survival was 59%.^[8]^

Histopathological evaluation, including immunohistochemistry for endothelial markers, is pivotal in confirming the diagnosis of EHE. The histologic features of EHE consist of epithelioid endothelial cells arranged in nests and cords that are surrounded by abundant myxoid or hyaline stroma. The tumor cells can be ovoid, cuboidal, or spindle-shaped with prominent eosinophilic cytoplasm, and some have intracytoplasmic vacuoles. Immunofluorescence staining is frequently required for definitive diagnosis, showing positivity for vascular markers including CD31, CD34, and FLI-1. A key molecular feature of EHE is the recurrent WWTR1-CAMTA1 gene fusion, which is present in approximately 90% of cases and is highly specific for EHE.^[5,9]^ This fusion can assist in distinguishing EHE from other vascular tumors, especially in diagnostically ambiguous cases. However, molecular testing is not always routinely available, and diagnosis still relies heavily on careful histopathological and immunohistochemical evaluation.

### 3.3. Therapeutic strategies and prognosis

Surgery is the treatment of choice for confirmed unifocal EHE, which tends to present in soft tissue. The sarcoma surgery guidelines should be followed for determining the extent of resection, and en bloc resection is necessary for a soft tissue EHE. To reduce the chance of local recurrence, R0 margins should be achieved by leaving a cuff of normal tissue surrounding the tumor surface. Radiation may be required as adjuvant therapy in cases of R1 excision.^[10,11]^ Given that EHE remains a rare malignancy, there is limited evidence guiding the use of adjuvant therapies. Given the propensity for metastasis to the liver and lungs, regular follow-up imaging of the abdomen and chest is warranted. Furthermore, close collaboration with the oncology and radiation oncology departments is essential for potential additional treatments. Recent therapeutic advances for EHE is targeted on WWTR1-CAMTA1 gene fusion, and investigation of targeted agents such as sirolimus (an mammalian Target of Rapamycin inhibitor) and anti-vascular endothelial growth factor therapies, which have showed effect in select conditions.^[9,12]^

Surgical excision remains the primary treatment modality for localized tumors. Recurrence may result from incomplete initial excision or misdiagnosis at the time of the first surgery, which happed in our case. After resection, multidisciplinary treatment with oncology and radiation oncology must be planned to minimize the potential of recurrence.

A case series reported that the median distant recurrence-free survival after resection of the primary tumor was 64.6 months for the 17 patients with localized EHE.^[13]^ The series authors also identified no prognostic factors for localized EHE. Another case series involving more than 150 patients with localized EHE revealed an 82% 5-year overall survival rate.^[14]^

### 3.4. Limitation and conclusions

Limitations of our work include the absence of molecular confirmation, and limited duration of postoperative follow-up. However, this case report highlights a rare incidence of EHE in the soft tissue, showing challenges in diagnostic workup and proper surgical management. A thorough clinical, radiological, and histopathological assessment contributed to the challenging diagnosis and subsequent management. As research advances, a deeper understanding of EHE pathogenesis and behavior will hopefully pave the way for more effective management strategies and improved patient outcomes.

## Acknowledgments

The authors wish to acknowledge the financial support of The Catholic Medical Center Research Foundation in the program year of 2024.

## Author contributions

**Data curation:** Jong Yun Choi, Suk-Ho Moon.

**Formal analysis:** Deuk Young Oh.

**Investigation:** Jong Yun Choi.

**Methodology:** Young-Joon Jun.

**Resources:** Jong Yun Choi.

**Supervision:** Jangyoun Choi.

**Validation:** Young-Joon Jun.

**Visualization:** Suk-Ho Moon.

**Writing – original draft:** Jong Yun Choi.

**Writing – review & editing:** Hee Yang Park, Deuk Young Oh, Jangyoun Choi.
